# How Blinded Soldiers Are Cared for

**Published:** 1917-01-20

**Authors:** Arnold Lawson

**Affiliations:** Ophthalmic Surgeon, Middlesex Hospital and St. Dunstan's.


### HOW BLINDED SOLDIERS ARE CARED FOR.

By MR. ARNOLD LAWSON, F.R.C.S., Ophthalmic Surgeon, Middlesex Hospital and St. Dunstan's.

In an address to the Medical Society of London in December last, Mr. Arnold Lawson, the well-known ophthalmologist, who has been associated with St. Dunstan's since its inception, gave many interesting particulars of the work that is being done there and of the principles upon which the treatment is carried on. The scheme, as most people are aware, owes its organisation and much of its success to the energy of Sir Arthur Pearson, himself afflicted with blindness whilst still no more than middle-aged. Early in 1915 it took shape in a small house for six patients in the Bayswater Road, from which small beginning it has grown to vast and ever-increasing dimensions.

### What St. Dunstan's has grown to.

Now the term "St. Dunstan's" stands for the main hostel in Regent's Park with its fifteen acres of land, where at the present moment live about 180 blinded soldiers, and where the training of them is mostly carried on. It also includes Regent's Park College, situated next door, so to speak, with its large grounds and accommodation for about 200 more men, which has generously been made over by the Baptist College Committee. Added to this are two large houses in Portland Place, which serve as accommodation for blind officers; whilst two others in Paddington are used for the lodgment of men for whom there is not available space elsewhere at the moment. Further, two convalescent homes, one at Brighton and one at Torquay, do extremely useful work in providing the men with week-end and longer trips to rebuild their health. These have been a great boon, as a number of the men suffer from headache and general malaise for months after their wounds have healed. Inasmuch as the work at St. Dunstan's does not finish with the actual training of the men, but purposes to settle them after training, to provide homes for them and to supervise them after settlement, an enormous work known as the "After-Care" Department has grown up and is constantly increasing. The actual settlement of the men is carried out by a special branch office with its clerks and officials at St. Dunstan's, whilst the after-care part is provided by apportioning the United Kingdom into eight districts each with its own organisation: one in Scotland, one in Ireland, and six in England. At the present moment there are 260 blinded sailors and soldiers at St. Dunstan's or its attendant convalescent homes at Brighton and Torquay. There are also 128 men in hospital either waiting for admission or sent into hospital for further treatment. During the twenty months of its life St. Dunstan's has fully trained and settled 125 blinded men.

### Classification and First Principles.

In the first place, the man is considered as blinded when his loss of sight is so severe as to render him under the ordinary circumstances of life incapable of carrying out an independent existence. The definition takes no count of those who have merely lost one eye; it includes not only those who are absolutely blind, but also those who have enough sight to find their way about, though it is insufficient to enable them to live independently of outside help. In this war the question of classification of a man as blinded has been greatly complicated by cases of what is technically termed "shell shock." It has been a most difficult point at St. Dunstan's what to do with some of these "shock" cases which are referred there for training as "blinded." The attempted differentiation of the functional from the organic element leads in not a few cases to a hopeless sense of bewilderment; but it suffices to say here that in a certain number it has been thought best to treat the men as though they were organically blind, and so incurable; whilst others have been referred to convalescent homes and other forms of treatment.

Secondly, it is important to realise that this war has already brought about a complete revolution in our ideas of training the blind. It is so, because for the first time in history we have been faced with a crowd of comparatively healthy, robust adults, mostly in the early days of their manhood, and suddenly deprived of their sight. Before the war the blind were mostly drawn from two sources: (1) The blind infants or very young children; and (2) the aged blind. The blind infants with no recollection of sight grew up under suitable tuition to take their place in the world, and of all blind classes have presented the least difficulty. The second class, the aged blind, formed and will always form a class for which very little training is applicable, and their position has always been and always will be the most pitiable of all.

### The Buoyancy of Youth.

The blind robust youth was until this war a comparative rarity—so rare that societies for the blind have found but little difficulty in coping with the number of cases requiring assistance. Now the burning question is the ever-increasing crowd of blind youths who at the outset of their careers are deprived of the means of combating the world. The sympathy of the world will ever, and naturally so, go out to the youthful sufferer rather than to the aged; but youth is characterised essentially by its buoyancy, its power of repair (mental as well as physical), and its inherent animal faculty of living in and for the present, without reference to a remote future. For those who are getting old it is different. The drab future stands out boldly in characters that cannot be misunderstood; the buoyancy of youth is gone, and the severed threads cannot be bound together again. There is an air of unforced cheerfulness at St. Dunstan's which is due to the youth of most of the occupants who, being youths, soon learn to realise that loss of sight does not mean for them the loss of all that makes life worth living.

That is the first lesson the men are taught. Self-pity is not permitted. Patting on the shoulder and commiseration with the poor fellow in the cruel hardness of his lot is not encouraged and is not done. He is taught to accept his lot as an inconvenience but not as a disability. He has got to make the best of it, and the best is a very good one when once he has been shown how. This lesson is learnt in a surprisingly short time when the surroundings of the youthful patient fit the case; and it precedes and leads up to the most important moral point of the whole training—viz., the teaching of the blinded man to recover and to preserve his independence.

### The Value of Independence.

St. Dunstan's has justified its work, Mr. Lawson believes, in no better way than in the teaching of independence. The inevitable consequence of losing sight is to cause an unconscious and increasing loss of the sufferer's independence. He leans more and more on others, and consequently gets more and more distrustful of his powers to do anything for himself.

This natural phase is most thoroughly discountenanced at St. Dunstan's. The new arrival is taken in hand by appointed members of the staff, and is carefully shown round the various rooms and workshops, and after some preliminary drilling is left to look after himself. Of course, there is always plenty of help handy all over the buildings should anyone need it at any time, and there are all sorts of simple devices to keep patients in the right way and prevent accidents. Thus the tracks all over the buildings are lined with linoleum, and the latter is bordered by carpet, the raised edge of which is easily distinguished by a stick. The man cannot do wrong if he walks on the linoleum track, and he distinguishes the turnings by the direction taken by the carpet. So, again, the presence of a door or steps is indicated by the insertion of some wooden planking before the obstacle is reached. The wood is at once appreciated, and the man instinctively slows up to meet the step or door. Nurses and orderlies are posted here and there at dangerous traffic corners, but no guide or helpers beyond a stick and a friendly hand from a pal or nurse is given or needed. It is, indeed, extraordinary to see how soon, by such simple methods, the blind man regains his lost confidence and cheerily goes about his own business without thought of outside help.

### The Mentality of the Blinded Soldier.

Another much more subtle difficulty is the natural "suspicion" of a blind man. Everybody instinctively gauges the value of a word or action as much by looking at the speaker or doer as by anything else. The judge or counsel in Court estimates the witness largely by watching him as he gives his evidence, and the loss of this faculty of watching lies very heavily on many blinded people. They become inherently suspicious of all strangers, and very apt in consequence to misconstrue and misunderstand in a way which frequently makes them most difficult to those who do not appreciate this point. This fact came out most strongly in St. Dunstan's in the early days when, for purposes of advertising and so helping on the place, visitors were freely welcomed and allowed to wander about the buildings and to converse with the men. It speedily became evident that the men strongly resented this, and the state of feeling was very pithily put by an ex-Irish Guardsman, who said, "I have been a member of the British Expeditionary Force; but now it appears I am a member of the British Exhibitionary Force."

The result was that it was found necessary to strictly regulate visitors and to post up notices asking that visitors should not speak to the men unless personally acquainted with them. It has been found that this common feeling of distrust and suspicion soon wears off in the life of good-fellowship and inspiration afforded by the experiences of those who have lived at the hostel for some time.

### Reading and Writing.

At the outset half the work-day is always given up to lessons in Braille and the typewriter. The former, many of the older patients, especially those who have passed their life in agricultural work, find very difficult. The delicacy of touch to make a reader sufficiently expert to enjoy reading as a pleasure is so great and requires to be kept up so assiduously, that it is to be feared that it is ultimately given up after a time by not a few. The typewriter, on the other hand, has proved a most satisfactory instrument, and the precision, speed, and accuracy of many of the men after a few weeks' coaching is remarkable. Every man at St. Dunstan's who succeeds in passing the test of efficiency in typing—and the test is no mean one—is presented on leaving the hostel with a typewriter.

The other half of the work-day is devoted to the teaching of handicrafts and trades. The selection of suitable employment is not always easy, and in a certain number of cases the unfitness of the man for something he has selected leads to the abandonment of this for something else. For what one might call the "higher occupations," for which only a selected number are obviously eligible, there are massage, poultry farming, and telephony; whilst the simpler handicrafts include mat-making, basket and hamper making, carpentering, and cobbling. These latter require less time, and it is customary to teach a man two or three handicrafts.

### Physical Fitness.

Then there is the difficult question of keeping the men "fit"—a most important point. The grounds of St. Dunstan's are a fortunate asset in this respect, and a certain time is given up in the mornings before breakfast to Swedish exercises carried out in true military fashion by a trained blind instructor. The physical exercises are voluntary, and in the winter are practised in the big hall. Then, too, as the grounds adjoin the park ornamental waters, rowing exercise is a very favourite pastime in the mornings before work begins. For the rest, plenty of voluntary helpers have come forward to take the men out for walking exercise, and altogether the physical side of the training is well cared for.

Lastly, after the training is completed, a process which usually means a residence of from seven to nine months, the man has to be "settled." The "settlement" and "after care" of the men is an enormous but most important part of St. Dunstan's work. The man is found a home in some suitable spot, and often his native heath is open to receive and help him, and there he is set up in business. That is his settlement. But beyond that he will always need the aiding hand of the "After-Care Department." A market must be found for his work, and his materials must be selected and the work itself supervised. A blind workman will make mistakes like a normally sighted workman, only with this difference—viz. that the blind man cannot see his mistakes and is very apt on this account to miss them, so that unless he is supervised the mistake becomes habitual and the quality of his work deteriorates. Further, to market his goods is difficult for him, for he may easily become the prey of the unscrupulous, whilst in a similar manner the materials for his work are best selected for him by somebody who sees that he gets value for his money. Thus the After-Care Department of St. Dunstan's is growing into a very large undertaking and is quite one of the most important features of the work.

